# Prolonged extracorporeal membrane oxygenation for pediatric necrotizing pneumonia due to *Streptococcus pneumonia* and influenza H1N1 co-infection: how long should we wait for native lung recovery?

**DOI:** 10.1007/s10047-018-1024-7

**Published:** 2018-02-05

**Authors:** Gerard Cortina, Christian Niederwanger, Uwe Klingkowski, Corinna Velik-Salchner, Nikolaus Neu

**Affiliations:** 1 Pediatric Intensive Care Unit, Department of Pediatrics, Medical University of Innsbruck, Innsbruck, Austria; 2 Department of Anesthesiology, Medical University of Innsbruck, Innsbruck, Austria; 3 Department of Pediatrics, Medical University of Innsbruck, Anichstrasse 35, 6020 Innsbruck, Austria

**Keywords:** Prolonged extracorporeal membrane oxygenation, Necrotizing pneumonia, Surgery, Native lung recovery

## Abstract

Most children with severe respiratory failure require extracorporeal membrane oxygenation (ECMO) for 7–10 days. However, some may need prolonged duration ECMO (> 14 days). To date, no consensus exists on how long to wait for native lung recovery. Here we report the case of a 3-year-old boy who developed severe necrotizing pneumonia requiring venovenous (VV) ECMO after 19 days of mechanical ventilation. In the first 4 weeks of his ECMO run, he showed no lung aeration, requiring total extracorporeal support. However, after we started strategies for promoting lung recovery such as daily prone positioning and regular use of toilet bronchoscopy and inhalative DNAse to clear secretions, by week five his tidal volumes gradually increased and he was successfully decannulated after 43 days. Moreover, we decided not to proceed to a surgical removal of the necrotic lung area. At present, he is 1-year post discharge and has fully recovered. This report shows that unexpected native lung recovery is possible even after prolonged loss of lung function and that a previous healthy lung can recover from apparent irreversible lung injury.

## Case report

**Fig. 1:**
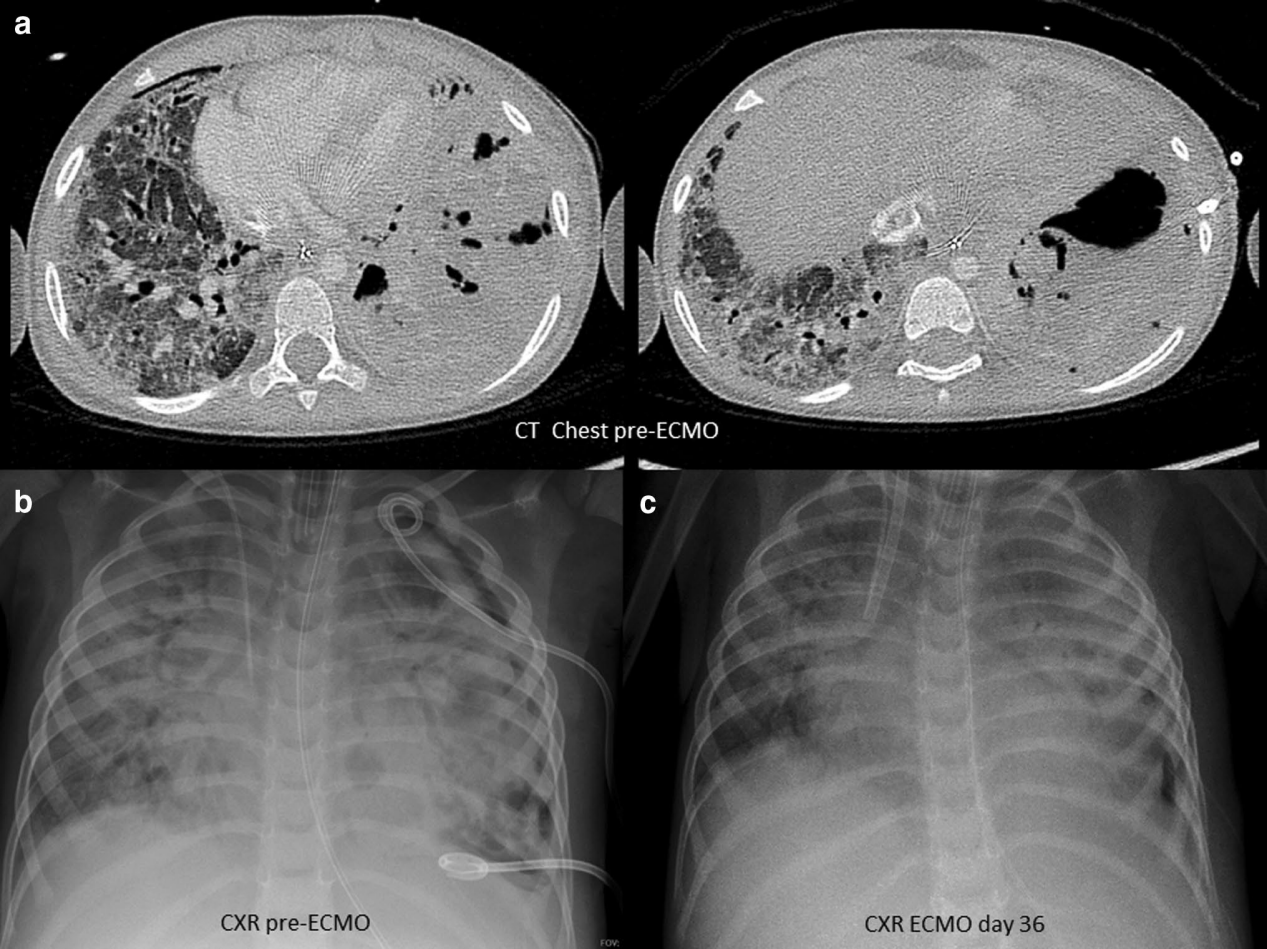
Chest computer tomography before ECMO and chest X-ray before and during ECMO

**Fig. 2:**
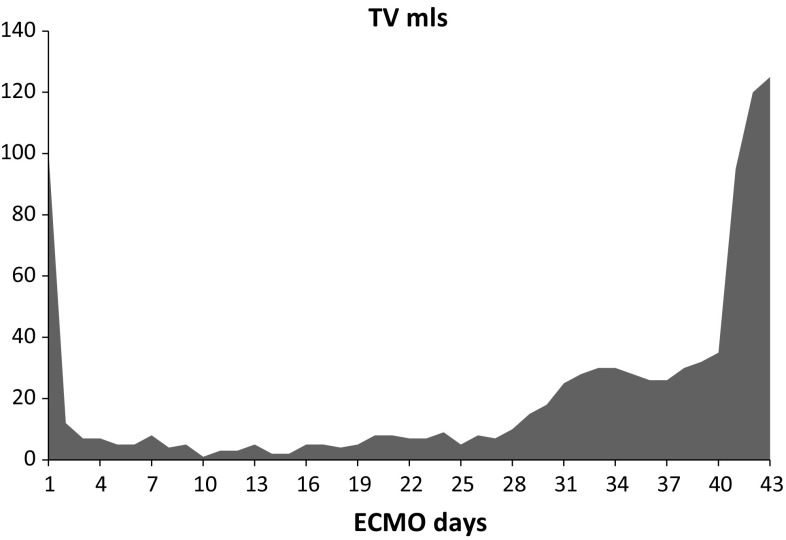
Tidal volumes during ECMO run

**Fig. 3:**
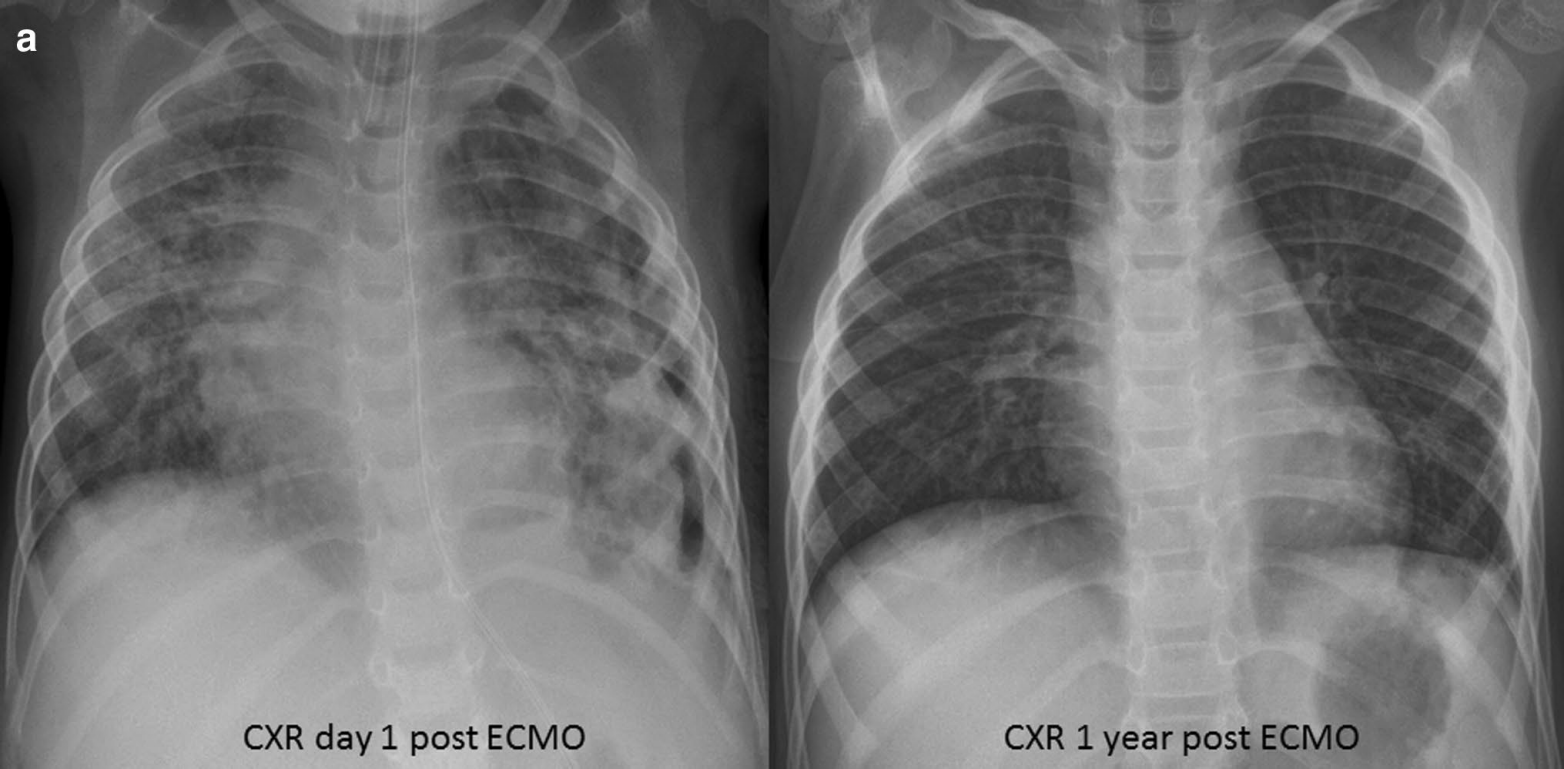
Chest X-ray after ECMO

A 3-year-old boy (body weight 17 kg) presented to a regional hospital with a 5-day history of cough and febrile illness. He was diagnosed with left-sided pneumonia and started on intravenous antibiotics (Ampicillin). The boy was previously healthy and up to date with his immunizations (including *Streptococcus pneumonia)*. Due to worsening respiratory distress he was transferred to our pediatric intensive care unit (PICU) the next day. He developed acute respiratory failure, followed by endotracheal intubation and invasive ventilation. The chest X-ray (CXR) showed an effusion and pneumothorax on the left side which required the placement of two chest drains. *Streptococcus pneumonia* was isolated from the chest drain fluid and Influenza H1N1 from a tracheal aspirate via polymerase chain reaction (PCR). A chest computer tomography (CT) showed a severe necrotizing pneumonia of the left lung with destruction of lung parenchyma and formation of a large abscess cavity in the lower lobe, as well as consolidation of the right lung (Fig. 1). A trial of high frequency ventilation and inhaled nitric oxide did not improve oxygenation and was abandoned. Despite ventilation with high peak inspiratory pressure (PIP = 40 cm H_2_O), high positive end-expiratory pressure (PEEP = 10 cm H_2_O) and FIO_2_ 100%, hypoxemia and hypercapnia became worse (oxygen saturation (SpO_2_) = 75–80%, arterial blood gas: pH = 7.28, paO_2_ = 38 mmHg, paCO_2_ = 127 mmHg), thus fulfilling the criteria for severe ARDS (PaO_2_/FiO_2_ = 38 mmHg, oxygenation index = 52.6) after excluding acute cardiac dysfunction. Consequently, venovenous extracorporeal membrane oxygenation (VV-ECMO) was implemented on day 19 of mechanical ventilation. A 15 French access cannula was placed into the left femoral vein and a 14 French return cannula into the right internal jugular vein. Initial ECMO settings were blood flow of 2 L/min, RPM 3000 and gas sweep of 1 L/min which immediately increased his SpO_2_ to 93%. Mechanical ventilation was reduced to lung rest settings (PIP 20 cm H_2_O, PEEP 10 cm H_2_O, respiratory rate 10/min, FIO_2_ 30%). Over the next 4 weeks our patient showed basically no lung aeration, depending completely on extracorporeal support. His tidal volumes (TV) were 5–8 mL, which is less than 0.5 mL/kg (Fig. 2). Additionally, due to insufficiency of the venous cannula we had to reduce flows and accepted SpO_2_ around 75% while keeping hemoglobin levels over 14 g/dL. Next, we started several measures to promote lung recovery, namely daily prone positioning for 12–16 h, twice daily inhalation with DNase and 2–3 toilet bronchoscopies per week to remove necrotic endobronchial material and to reduce its viscosity. This led to an increase in TV to 35 mL (2 mL/kg) by week five, but finally to 100 mL (6 mL/kg) on day 41, together with areas of re-aeration on CXR. Forty-eight hours later our patient was successfully decannulated (total VV-ECMO time 43 days). During his entire ECMO run, our patient was in single-organ system respiratory failure without exhibiting any serious complications. Over the next 3 weeks, ventilation was slowly weaned as his native lung function continued to improve (total mechanical ventilation time 88 days). He was subsequently discharged from PICU and home 4 weeks later without additional oxygen. Because we hesitated to expose him to the risk of a both general anesthesia and extended surgery, we decided not to proceed to a surgical resection of the necrotic area, although the large abscess was still visible on repeat chest CT. Our patient is now 1 year post discharge and has normal oxygen saturation in room air and has fully recovered. His latest CXR showed only small residual parenchymal changes and a thickening of the pleura of the left lung (Fig. 3).

## Discussion

ECMO is a well-established rescue therapy for refractory respiratory failure. Historically, ELSO registry data suggest a survival rate to discharge of 57% for pediatric respiratory ECMO, including all causes of respiratory failure [1]. A single-center evaluation of patients with refractory pneumonia supported with ECMO showed overall survival rates to discharge of 69.2% over a 23-year period and 88.2% in the era after 2005. Median ECMO duration in this cohort of 52 patients was 7.35 days [2]. Prolonged ECMO duration has been defined as > 14 days and is increasingly being reported. However, there is a lack of experience with long-term extracorporeal pulmonary support due to the scanty literature. An ELSO registry study of pediatric ECMO for respiratory support showed that children supported for ≥ 21 days had a significantly lower survival than those supported for < 14 days (38% vs. 61%) and the authors concluded that survival declines with duration of ECMO [3]. A single-center study of 951 children treated with ECMO for cardiac and respiratory support over a period of 20 years (1991–2011) concluded that prolonged ECMO use was associated with low survival [4]. In this series, only 22 ECMO runs were ≥ 28 days, and of these only four patients (19%) survived to hospital discharge. Another recent adult ELSO (extracorporeal life support organization) registry study showed that approximately one out of five adult patients required prolonged ECMO for respiratory failure and that survival rate to hospital discharge in these patients was 45.4% and about 10% lower than in patients with shorter ECMO runs [5]. Taken together, it seems that ECMO duration has a negative impact on survival. However, we felt encouraged by previous successful case reports on prolonged ECMO use for influenza H1N1-associated respiratory failure, and given that our patient was in single-organ failure and did not encounter any complications of extracorporeal support, we decided to continue ECMO [6]. Moreover, the ELSO registry study showed that, although survival declined with increasing ECMO duration, it still exceeded 30% after 4 weeks of ECMO [3]. Longer pre-ECMO ventilation times have also been associated with decreased survival as a result of ventilator induced lung injury. Historically, accepted pediatric criteria for patient selection included mechanical ventilation < 7–10 days [7]. A more recent ELSO registry report found that there was no statistically significant decrease in survival until > 14 days pre-ECMO mechanical ventilation was reached [8]. In cases like ours with significant lung destruction caused by *Streptococcus pneumonia* and H1N1 co-infection and aggravated by 19 days of mechanical ventilation pre-ECMO, it is possible that those children need more extended support to allow for eventual native lung recovery. We believe that our case offers some unique aspects. First, strategies for promoting lung recovery while on ECMO have not been well studied. We observed improvement in oxygenation only after we started daily prone positioning together with increased secretion clearance by means of inhalation with DNase and regular toilet bronchoscopies. Second, we applied the concept of permissive hypoxemia, aiming for SpO_2_ around 75%, to allow ECMO flow reductions. This was well tolerated over a prolonged period of time while keeping the Hb > 14 g/dL. Third, we observed a complete recovery of the lung parenchyma without surgery, although most of the lower lobe of the left lung was necrotic.

To summarize, at present we are unable to predict who will survive respiratory ECMO and who will not. Therefore, some groups recommend to keep ECMO running as long as necessary in patients with single-organ system respiratory failure. The lung may have unexpected regenerative capacity and time to native lung recovery may be longer than expected in some patients. Additionally, even in cases with significant lung destruction, surgery might not always be a good choice. Our case demonstrates that current technological advances in ECMO circuit allow prolonged support without complications and that strategies for promoting lung recovery while on ECMO should be further evaluated.
